# Future L2 writing selves and EFL writing achievement: the mediating role of writing enjoyment and boredom

**DOI:** 10.3389/fpsyg.2026.1802306

**Published:** 2026-04-24

**Authors:** Jie Pan, Huimei Chen

**Affiliations:** 1 Shanghai Normal University Tianhua College, Shanghai, China; 2 College of Education, Northern Arizona University, Flagstaff, AZ, United States

**Keywords:** EFL writing boredom, EFL writing enjoyment, future L2 writing selves, mediating effects, writing achievement

## Abstract

**Introduction:**

Second language (L2) writing is not only a cognitive activity but also an emotionally involving process. However, the joint influence of motivational self-guides and achievement emotions on L2 writing outcomes remains underexplored in Asia-Pacific contexts. Drawing on the L2 Motivational Self System and Control-Value Theory, this study examined how future L2 writing selves predict English as a Foreign Language (EFL) writing achievement through foreign language writing enjoyment (FLWE) and boredom (FLWB).

**Methods:**

This quantitative cross-sectional study involved 349 first-year Chinese EFL undergraduates. Data were collected through validated self-report scales and a standardized argumentative writing task. Confirmatory factor analysis (CFA) and parallel multiple mediation analysis (PROCESS Model 4) were conducted.

**Results:**

Both the ideal L2 writing self (ILWS) and the ought-to L2 writing self (OLWS/own) positively predicted FLWE and writing achievement, and negatively predicted FLWB. FLWE positively predicted writing achievement, whereas FLWB negatively predicted it. FLWE and FLWB jointly and partially mediated the relationships between future L2 writing selves and writing achievement, with enjoyment emerging as the stronger mediator.

**Discussion:**

These findings highlight the dynamic interplay between motivation and emotion in exam-oriented EFL settings and underscore the importance of fostering future-oriented writing identities alongside positive emotional experiences to enhance L2 writing performance.

## 1 Introduction

Writing is a cognitive, goal-oriented, and participatory activity that requires considerable mental effort and is significantly influenced by emotional, motivational, and attitudinal factors (Hayes, 1996). Although the cognitive mechanisms underlying second language (L2) writing have been extensively examined, considerably less attention has been paid to the motivational and emotional factors in the writing process (Li et al., 2023; Tahmouresi and Papi, 2021). This imbalance is noteworthy, as writing is not merely a cognitive act but also an emotionally involving activity in which learners must cope with cognitive demands and linguistic challenges while continuously evaluating their competence, goals, and progress (Li et al., 2023). In foreign language (FL) writing contexts, motivation and emotion are likely to operate jointly rather than independently, shaping learners’ persistence, engagement, and ultimately, achievement (Jang and Lee, 2019). A more integrated account of these psycho-affective processes is therefore needed.

Motivation has long been recognized as a central driving force in L2 learning (Dörnyei and Ryan, 2015). In writing contexts, motivation is manifested in sustained task engagement (Tsao et al., 2017), proactive feedback-seeking behaviors (Papi et al., 2020), and improved writing performance (Tahmouresi and Papi, 2021; Yao et al., 2024). However, despite its acknowledged importance, the mechanisms through which motivational dispositions translate into writing achievement remain underexplored (Jang and Lee, 2019). Much of the existing literature has focused on broad motivational orientations without sufficiently examining how future-oriented self-guides may shape learners’ emotional experiences during writing. This issue is particularly salient in the Chinese tertiary EFL context, where students often navigate a dual motivational landscape—balancing personal aspirations with strong academic and social expectations. Understanding how such future self-concepts operate within writing tasks may provide clearer insight into how motivation becomes behaviorally and academically consequential.

Emotion constitutes another decisive component in the L2 writing process and its outcomes. Li et al. (2023) emphasized that L2 writers are “emotionally responsive” to writing tasks, experiencing both positive and negative emotional states. Nevertheless, empirical research on L2 writing emotions has long centered on writing apprehension and writing anxiety, both of which occupy an important and well-established place in the literature (e.g., Cheng, 2004; Teimouri et al., 2019). In general, learners with high levels of writing apprehension tend to experience anxiety in writing situations, expect themselves to fail, fear evaluation of their writing, and show a tendency to avoid writing tasks (Daly and Miller, 1975a,b; Smith, 1984). Other prevalent achievement emotions—such as foreign language writing enjoyment (FLWE) and boredom (FLWB)—have, by comparison, received comparatively limited attention. The present study does not seek to challenge the importance of the writing apprehension/anxiety tradition; rather, it deliberately focuses on enjoyment and boredom in order to extend the emotional landscape of L2 writing research beyond its longstanding emphasis on anxiety-related constructs. Initial evidence from Li et al. (2023) suggests that boredom may be an even stronger negative predictor of writing achievement than anxiety, whereas enjoyment functions as a positive predictor. At the same time, boredom should not be treated as uniformly maladaptive in every instance. It may also function as an affective signal that a learner’s current engagement with the task is suboptimal, and under some conditions this may prompt effort regulation or strategic adjustment (Danckert et al., 2018). From a control-value perspective, boredom may arise when tasks are perceived as either far beyond or below learners’ capabilities, suggesting that at least some of its antecedents may be manageable through modifications in task design, challenge level, or instructional support (Tze et al., 2016). Despite these important findings, motivational variables were not incorporated into Li et al.’s (2023) model. Similarly, although Tahmouresi and Papi (2021) examined the joint effects of motivational and emotional factors, their focus was primarily on enjoyment and anxiety, leaving boredom understudied. This omission is theoretically consequential. Li and Han (2022) argued that boredom serves as a proximal counterpart to enjoyment within the control-value framework, representing a distinct yet equally powerful predictor of engagement and academic attainment in L2 writing. Given that L2 writing requires learners to manage cognitive demands and linguistic challenges and may become repetitive across writing tasks, boredom may exert a particularly direct influence on writing engagement and performance (Li et al., 2023).

To address these gaps, the present study integrates Dörnyei’s (2005, 2009) L2 Motivational Self System (L2MSS) with Pekrun’s (2006) Control-Value Theory (CVT) to construct an integrated explanatory framework. Within this model, future L2 writing selves (FL2WS)—including the ideal L2 writing self (ILWS) and the ought-to L2 writing self (OLWS)—are conceptualized as distal motivational antecedents that shape learners’ proximal emotional experiences (FLWE and FLWB), which in turn influence writing achievement. In the present study, FL2WS is used as a superordinate umbrella term referring jointly to the ILWS and the OLWS, rather than as a replacement for Dörnyei’s (2009) original construct label ideal L2 self. By examining these interrelations among Chinese university EFL learners, the study seeks to clarify how future-oriented motivational self-guides are translated into concrete academic outcomes through emotional mechanisms. Such an approach contributes to a more comprehensive understanding of the psycho-affective architecture underlying L2 writing and responds to recent calls for integrative models that bridge motivation and emotion in applied linguistics research. The study is guided by the following research questions:

*RQ1:* How do FL2WS (ILWS and OLWS) predict FLWE, FLWB, and FL writing achievement?

*RQ2:* How do FLWE and FLWB predict FL writing achievement?

*RQ3:* Are the relationships between FL2WS and FL writing achievement mediated by FLWE and FLWB?

## 2 Literature review

Drawing upon the socio-educational model of motivation (Gardner, 1985), the theory of possible selves (Markus and Nurius, 1986), and self-discrepancy theory (Higgins, 1987), Dörnyei (2005) proposed the L2 Motivational Self System (L2MSS), which consists of three interrelated components: the ideal L2 self, the ought-to L2 self, and the L2 learning experience. The ideal L2 self reflects the extent to which learners envision themselves as competent users of the target language, while the ought-to L2 self pertains to the external obligations, expectations, and pressures that shape their motivation throughout the learning process. The third component, the L2 learning experience, involves context-specific motives rooted in the immediate learning environment, such as teaching methods, classroom atmosphere, and instructional resources.

Acknowledging certain limitations within L2MSS, Papi et al. (2019) introduced a “2 × 2 model” that intersects two regulatory focuses (ideal vs. ought-to) with two perspectives (own vs. other). This model distinguishes between self-generated aspirations (ideal/own) and externally imposed hopes (ideal/other), alongside a parallel distinction between internally endorsed responsibilities (ought-to/own) and externally mandated obligations (ought-to/other). In the field of L2 writing research, Tahmouresi and Papi (2021) sought to explore the future L2 writing selves of 48 Iranian English major university students. They found that only the ideal L2 writing self/own and the ought-to L2 writing self/own emerged as significant, with the ideal L2 writing self/others and ought-to L2 writing self/others notably absent. This omission possibly occurred because “the open-ended questions did not ask the students to list their important others’ (e.g., parents) hope and obligations related to their English writing skills” (p. 5). Following Tahmouresi and Papi (2021), the term future L2 writing selves is used here as an umbrella label for the two future-oriented writing self-guides examined in this study. Accordingly, the present study specifically focuses on the ideal L2 writing self/own (ILWS/own) and the ought-to L2 writing self/own (OLWS/own). According to Tahmouresi and Papi (2021), ILWS represents learners’ personal aspirations to master foreign language writing skills, whereas OLWS refers to the writing abilities learners believe they should possess to fulfill personal expectations and avoid potential negative consequences.

The predictive capacity of these selves, however, remains inconsistent. Specifically, ILWS consistently serves as a robust predictor of L2 achievement and positive emotional experiences (e.g., Tahmouresi and Papi, 2021; Yao et al., 2024). Conversely, the relationship between OLWS and these outcomes has been ambiguous, with numerous studies reporting weak or null findings (Al-Hoorie, 2018; Jang and Lee, 2019). This inconsistency raises a critical theoretical question: Has the potential of the ought-to self been underestimated due to a lack of distinction between externally imposed pressures and internally endorsed responsibilities? This study argues that the OLWS/own warrants renewed investigation, particularly within the relatively underexplored context of EFL writing.

While motivational factors are pivotal, L2 writing is equally an emotional endeavor. Research on emotions in foreign language (FL) writing has traditionally been shaped by the well-established constructs of writing apprehension and anxiety, which have made an important contribution to understanding the affective dimension of writing. At the same time, other writing-related achievement emotions have received comparatively less attention. Pekrun's (2006) Control-Value Theory (CVT) offers a comprehensive framework for addressing this imbalance. CVT categorizes achievement emotions based on their valence (positive or negative) and activation (activating or deactivating). Within this taxonomy, foreign language writing enjoyment (FLWE) is characterized as a positive, activating emotion that may stem from personal satisfaction (private enjoyment) or a supportive classroom environment (social enjoyment) (Li et al., 2023). Conversely, foreign language writing boredom (FLWB) is a negative, deactivating emotion associated with low interest, reduced attentional investment, and disengagement from writing tasks. At the same time, boredom should not be treated as uniformly maladaptive in every instance. Under some conditions, it may also function as a signal that the task is insufficiently stimulating, poorly matched to learners’ perceived competence, or in need of strategic adjustment. In writing-specific contexts, however, existing evidence has more consistently linked higher boredom to weaker engagement and less favorable performance outcomes (Li et al., 2023; Li and Li, 2024).

Research on general language learning indicates that enjoyment enhances achievement, whereas boredom undermines it (e.g., Botes et al., 2022; Li and Li, 2023). However, these generalized findings may obscure the distinct emotional dynamics related to specific skills such as writing. As a cognitively demanding and frequently personal activity, writing poses unique affective challenges that standard general emotion measures may overlook (Cheng, 2004; Teimouri et al., 2019). Indeed, the association between a general emotion and a skill-specific emotion can be tenuous. For instance, Dewaele et al. (2023) found no significant impact of general foreign language boredom on overall achievement, which contrasts sharply with the negative role of boredom identified in domain-specific studies. This discrepancy underscores a crucial point: to fully comprehend the factors influencing writing performance, it is essential to analyze the emotions inherently tied to the writing process itself.

To empirically capture these writing-specific affective states, Li et al. (2023) developed scales to measure FLWE and FLWB. Their research established that, in the context of writing, these emotions serve as significant predictors: FLWE positively influences writing achievement, while FLWB adversely affects it. This finding was subsequently validated by Li and Li (2024) in a more extensive study. Therefore, the rationale for examining emotions specific to writing is robust. Nevertheless, a significant question remains largely unexplored: how do these two contrasting emotions—enjoyment and boredom—operate concurrently when incorporated into a unified model of L2 writing? The simultaneous examination of their joint impact constitutes an essential progression in the existing literature.

The relationship between future self-images and immediate emotional experiences is not coincidental; it is theoretically substantiated. Higgins’s (1987) self-discrepancy theory, along with its extension, regulatory focus theory (Higgins, 1997, 1998), provides a robust framework for understanding these psychological phenomena. These theories posit that the ideal self is associated with a promotion focus, where individuals are driven by aspirations and gains, making them acutely aware of whether favorable results are occurring. In contrast, the ought-to self is linked to a prevention focus, where the primary motivation revolves around safety and responsibility, leading individuals to be attuned to the presence or absence of negative outcomes.

This theoretical paradigm helps elucidate the empirical record. ILWS, functioning as a promotion-focused guide, consistently cultivates positive, activating feelings such as enjoyment when progress is achieved (Le, 2024; Liu et al., 2022; Papi and Khajavy, 2021; Teimouri, 2017). Additionally, a negative association between ILWS and boredom is becoming increasingly evident in the literature. For example, Csizér et al. (2021) discovered a distinct negative correlation between the ideal L2 self and boredom, a conclusion supported by Tang et al. (2024) and Amini et al. (2023), who validated that the ideal L2 self serves as a negative predictor of boredom. This aligns with the premise that learners who are focused on making progress are intrinsically motivated to overcome or eliminate boring situations (Struk et al., 2016).

The dynamics surrounding OLWS are more intricate and appear to depend significantly on the differentiation between one’s own perspective and that of others. Utilizing the 2 × 2 model, Amini et al. (2023) demonstrated that the ought-to L2 self/other positively correlates with boredom and negatively correlates with enjoyment. This reflects the influence of externally imposed expectations, such as parental demands or institutional requirements, and the perceived need to avoid negative consequences (e.g., poor academic performance or social disapproval). Within the framework of regulatory focus theory (Higgins, 1997, 1998), such externally regulated ought-to selves are typically aligned with a prevention focus, whereby individuals become highly vigilant toward the avoidance of losses and failures. This prevention-oriented self-regulation heightens sensitivity to obligation and external evaluation, which may increase tension and disengagement, thereby explaining its positive association with boredom.

The primary theoretical proposition presented here is that when ought-to demands are internalized—specifically, when they transition into OLWS/own—their functional characteristics may shift, evolving from an external compulsion into a personal criterion for self-regulation. In accordance with self-discrepancy theory (Higgins, 1987), motivational force arises from perceived discrepancies between the actual self and one’s self-guides. When environmental expectations are internalized, this discrepancy is no longer experienced purely as an externally imposed pressure, but rather as a personally meaningful standard to be attained, thereby strengthening self-regulatory commitment. The findings of Papi et al. (2019) support this perspective, indicating that when learners recognize the possibility of significant negative consequences, the ought-to L2 self can serve as a powerful self-guide, strategically influencing behavior to avoid such unfavorable outcomes.

The process of this internalization is illuminated by Csizér and Lukács (2010), who observed that the ought-to L2 self develops as learners mature and start to “internalize the pressure the environment might put on them” (p. 6), gradually integrating external expectations into their evolving self-concept. From a regulatory focus perspective (Higgins, 1997, 1998), such internally endorsed ought-to standards remain prevention-oriented, as they are still sensitive to potential losses or failures; however, once internalized, this prevention-focused regulation may operate in a more autonomous and strategic manner. It facilitates effort regulation and adaptive engagement rather than mere passive compliance. In the present study, this internalization implies that learners may come to view effective writing not merely as a teacher- or parent-imposed obligation, but as a self-relevant responsibility deeply embedded within their academic identity.

Supporting this mechanism, Le (2024) identified a significant, albeit modest, positive correlation between the ought-to L2 self and learning enjoyment in the context of foreign language vocabulary acquisition. This underlying process, in which internalized pressure promotes strategic engagement, may also apply robustly to L2 writing contexts. Learners may adopt the responsibility of writing effectively as a personal commitment, actively utilizing strategies to make the writing process more manageable and enjoyable. This, in turn, reduces boredom and sustains effort (Struk et al., 2016; Smith et al., 2009). Consequently, OLWS/own may function fundamentally differently from externally controlled ought-to selves, potentially serving as a significant predictor of increased enjoyment and decreased boredom in high-stakes contexts—a hypothesis that this study directly examines.

The preceding synthesis underscores that prospective L2 writing identities serve as salient predictors of both emotional responses to FL writing and subsequent writing achievement. However, a pivotal inquiry persists: through what precise mechanism do these motivational self-guides translate into tangible writing outcomes? We propose that the answer lies in the mediating effects of achievement emotions. Although the individual component connections in this causal chain—from FL2WS to emotions, and from emotions to achievement—have garnered empirical validation, the comprehensive pathway remains insufficiently delineated.

Current research provides preliminary yet markedly inadequate data regarding this mediation mechanism. For example, while multiple studies have substantiated that Foreign Language Enjoyment (FLE) can mediate the relationship between the ideal L2 self and overall achievement in general foreign language learning (e.g., Dörnyei and Chan, 2013; Fathi and Hejazi, 2024; Jiang and Papi, 2022) as well as in the specific area of writing (e.g., Tahmouresi and Papi, 2021; Zhang et al., 2025; Zhu et al., 2022), findings on this issue have not been fully consistent across contexts. Yao et al. (2024), for example, reported that in the high-pressure setting of Chinese senior high schools, FLWE failed to directly predict writing achievement and therefore did not function as a meaningful mediator. Considering that FLWE and FLWB possess conflicting valence and activation (Pekrun, 2006), a comprehensive understanding requires an examination of how ideal selves can simultaneously mitigate FLWB and, under conducive conditions, enhance FLWE.

Furthermore, the potential for a parallel mediation pathway concerning the requisite OLWS/own has been largely overlooked. The theoretical framework established earlier posits that OLWS/own, functioning as an internalized prevention-oriented guide, should also impact academic accomplishment by influencing students’ emotional experiences within the writing classroom. If OLWS/own genuinely encourages students to take charge of their learning environment to avoid failure, this autonomous effort should reflect in heightened enjoyment and diminished boredom, ultimately facilitating better performance. To date, no research has examined this proposed emotional pathway for OLWS/own.

In conclusion, the current study aims to address these interrelated gaps in the literature by presenting and evaluating a parallel multiple mediation model. This model systematically asserts that both FLWE and FLWB function as mediating mechanisms in the relationship between FL2WS (i.e., ILWS and OLWS) and FL writing achievement.

## 3 Methodology

### 3.1 Context and participants

The study was conducted at a private university in eastern China. Unlike public institutions, private colleges in China primarily rely on tuition and funding from private entities while receiving limited governmental support (Su, 2012), and they generally emphasize economic sustainability and returns on investment (Li and Morgan, 2008). Freshmen are assigned to administrative classes based on their Gaokao performance and major, with each class typically accommodating around 35 students. The university has established Sino-foreign collaborative programs with several American institutions across multiple disciplines.

Participants were recruited through convenience sampling due to accessibility within the institutional setting (Creswell and Creswell, 2017). Although this approach limits broader generalizability, it is widely adopted in educational research when relying on intact instructional groups (Etikan et al., 2016). The study focused on first-year undergraduates enrolled in compulsory College English Writing courses within collaborative programs. The writing course is a required component for students majoring in Rehabilitation Science (*N* = 98, 28.03%), Elementary Education (*N* = 92, 26.36%), Early Childhood Education (*N* = 86, 24.64%), and International Business (*N* = 73, 20.92%), providing a shared curricular context. In these majors, the English writing course is scheduled during the spring semester of the first academic year, whereas in other programs the writing course is offered during the fall semester of the first academic year. To ensure instructional consistency and comparable exposure to the writing curriculum, the present study focused exclusively on majors in which the course was delivered during the same semester. Recruitment was therefore conducted across all eligible majors offering the spring-semester writing course to secure an adequate and contextually homogeneous sample. While the sample was drawn from a single private institution and therefore cannot be considered statistically representative of all Chinese private university EFL learners, it included students from multiple majors within the same institutional and curricular framework. The relatively large sample size (*N* = 349) enhances statistical power and supports the examination of structural relationships among the focal variables within this defined educational context. Accordingly, the findings should be interpreted as context-specific rather than nationally representative.

A total of 349 EFL learners participated voluntarily. The gender distribution was 89 males (25.5%) and 260 females (74.5%). The average age of the participants was 19.25 years. All participants were Chinese nationals and native speakers of Mandarin Chinese. As displayed in Table 1, the mean score on the university’s post-enrollment English placement test was 71 out of 100 (*SD* = 12.10), indicating an intermediate level of general English proficiency. Participants’ self-perceived English proficiency was 5.51 (*SD* = 1.62) on a 10-point scale, which is slightly above the midpoint and suggests a moderate but somewhat cautious self-evaluation relative to their placement-test performance. Student identification numbers were collected solely for matching questionnaire responses with official final writing examination scores provided by course instructors, and all identifying information was anonymized prior to analysis.

**Table 1:** Descriptive statistics of participants’ background variables (*N* = 349).

Variables	Mean	SD	Range
Age	19.25	0.44	18–20
Years of learning English	13.13	1.87	6–15
Self-perceived English level	5.51	1.62	1–10
English placement test score	71.00	12.10	35–92

### 3.2 Instruments

This study employed a structured questionnaire consisting of two sections. The first section collected participants’ demographic information, including cohort, sex, age, major, years of learning English, self-perceived English proficiency level and post-enrollment English placement test (Table 1). Participants were required to provide their student identification numbers for research purposes only. These identifiers were used to match questionnaire responses with official final writing examination scores supplied by course instructors. All identifying information was anonymized prior to analysis. The second section measured students’ future L2 writing selves, foreign language writing enjoyment, and foreign language writing boredom. The future L2 writing selves scale (Tahmouresi and Papi, 2021) was administered in Chinese using a translation and back-translation procedure to ensure semantic equivalence. By contrast, the Foreign Language Writing Enjoyment and Boredom scales were originally developed and validated in Chinese (Li et al., 2023) and were therefore used in their original language version. Construct validity of the measurement models was examined via confirmatory factor analysis (CFA) in AMOS, and reliability was evaluated using Cronbach’s *α* and composite reliability (CR). Convergent validity was assessed by average variance extracted (AVE). Model fit was evaluated using *χ*^2^/df, CFI, TLI, RMSEA, and SRMR.

#### 3.2.1 Future L2 writing selves scale

The future L2 writing selves scale (Tahmouresi and Papi, 2021) was used to assess students’ ideal L2 writing self (ILWS) and ought-to L2 writing self (OLWS), with 5 items for each dimension. All items were rated on a six-point Likert scale ranging from 1 (strongly disagree) to 6 (strongly agree). A sample item for ILWS is “*I can see a day that I can easily write down my thoughts and ideas in English*,” whereas a sample item for OLWS is “*I must learn how to write in English; otherwise, I won’t be able to get my degree*.”

Confirmatory factor analysis (CFA) was conducted to examine construct validity. The ILWS model demonstrated acceptable fit (*χ*^2^/df = 3.285, CFI = 0.987, TLI = 0.975, RMSEA = 0.081, SRMR = 0.022), while the OLWS model showed excellent fit (*χ*^2^/df = 1.829, CFI = 0.995, TLI = 0.989, RMSEA = 0.049, SRMR = 0.018). Internal consistency reliability was satisfactory (ILWS *α* = 0.889; OLWS *α* = 0.866). Composite reliability values exceeded the recommended threshold of 0.70 (ILWS CR = 0.889; OLWS CR = 0.868), and AVE values were above 0.50 (ILWS AVE = 0.616; OLWS AVE = 0.568), indicating adequate convergent validity.

#### 3.2.2 The foreign language writing enjoyment scale

The Foreign language writing enjoyment scale (Li et al., 2023) was employed to measure students’ FLWE. The scale consists of two dimensions: Private Writing Enjoyment (PWES; 6 items) and Social Writing Enjoyment (SWES; 3 items). All items were rated on five-point Likert scales. A sample item for PWES is “*I enjoy putting what I have learned into English writing*,” and a sample item for SWES is “*We are always encouraged to write more in English by the English teacher*.”

CFA results indicated good model fit for PWES (*χ*^2^/df = 1.892, CFI = 0.985, TLI = 0.979, RMSEA = 0.051, SRMR = 0.028). Internal consistency was high (*α* = 0.852), and composite reliability reached 0.861. The AVE value (0.514) exceeded the recommended criterion of 0.50, supporting adequate convergent validity. The SWES subscale also demonstrated satisfactory reliability (*α* = 0.798; CR = 0.800) and acceptable convergent validity (AVE = 0.572).

#### 3.2.3 The foreign language writing boredom scale

The foreign language writing boredom scale (FLWBS), developed by Li et al. (2023), consists of five items measuring students’ boredom experiences in L2 writing. A sample item is “I find it difficult to feel engaged in English writing.” All items were rated on five-point Likert scales.

The CFA results indicated acceptable model fit (*χ*^2^/df = 2.911, CFI = 0.967, TLI = 0.934, RMSEA = 0.074, SRMR = 0.034). Internal consistency reliability was adequate (Cronbach’s *α* = 0.721), and composite reliability exceeded the recommended threshold (CR = 0.723). The AVE value (0.344) was below the conventional 0.50 criterion. However, all standardized factor loadings were statistically significant and model fit indices were within acceptable ranges. According to Fornell and Larcker (1981), convergent validity can still be considered adequate when composite reliability is above 0.60 despite a relatively low AVE. Similarly, Hair et al. (2010) noted that in social science research, AVE values slightly below 0.50 may be acceptable when CR exceeds 0.70 and the overall measurement model demonstrates satisfactory fit. Therefore, the FLWB scale was retained for subsequent analyses.

#### 3.2.4 Foreign language writing achievement

Foreign language writing achievement was assessed through a standardized end-of-semester examination. Students were required to complete a timed argumentative essay on the topic: “*Write an essay about the importance of developing a healthy lifestyle among college students*.” The task required approximately 180–200 words within 30 min. The prompt was developed in alignment with the College English Test Band 4 (CET-4) writing framework and was designed to elicit students’ ability to articulate a clear thesis, construct logically developed arguments, provide relevant supporting examples, and demonstrate coherence, lexical range, and grammatical control.

Prior to the examination, students completed a 12-week writing course consisting of four instructional periods per week (40 min each; total instructional time = 32 h). Instruction systematically addressed argumentative writing conventions, including thesis development, paragraph organization, logical progression, cohesion strategies, counterargument construction, and revision techniques. All sections followed a unified syllabus and synchronized pacing to ensure instructional consistency. Multiple guided and timed practice essays were completed to familiarize students with the task format and assessment criteria.

Essays were evaluated using an analytic rubric adapted from the CET-4 scoring framework. The rubric assessed task response, organization and coherence, lexical resource, and grammatical range and accuracy. Domain scores were aggregated into a total score ranging from 0 to 100.

Three native English-speaking instructors with more than 5 years of experience teaching academic writing in higher education served as human raters. All raters held postgraduate qualifications in TESOL or Applied Linguistics and participated in a calibration session prior to scoring to ensure alignment in rubric interpretation and scoring standards. To enhance scoring reliability and mitigate potential human bias, essays were independently evaluated using DeepSeek under the same analytic rubric and scoring protocol. The AI system operated on anonymized scripts and had no access to student-identifying information.

Inter-rater reliability between human and AI ratings was examined using a two-way random-effects intraclass correlation coefficient (absolute agreement), an approach appropriate when raters are considered representative of a broader rating population and when exact agreement between ratings is required (Koo and Li, 2016). The single-measure ICC was 0.869 [95% CI (0.552, 0.942)], indicating good agreement. Because the final score was derived from the combined ratings, the average-measures ICC (*k* = 2) is of primary relevance. The average-measures ICC was 0.930 [95% CI (0.712, 0.970)], reflecting excellent reliability for the averaged score (Koo and Li, 2016).

To ensure scoring fairness and minimize potential discrepancies, a predefined discrepancy resolution procedure was implemented. When the absolute difference between the teacher score and the DeepSeek score was within five points, the final score was calculated as the mean of the two ratings. When the discrepancy exceeded five points, the script was subjected to a review process in which the instructors discussed the case and reached a consensus score acceptable to all raters. This consensus score was then recorded as the final writing achievement score. Final scores ranged from 0 to 100 and were used as the outcome variable in subsequent analyses.

### 3.3 Data collection

Data collection started after the current research was approved by the Institutional Review Board (IRB) at Northern Arizona University. Subsequently, we reached out to the international English instructors and successfully obtained their approval and assistance. All essays were evaluated using the same analytic rubric adapted from the CET-4 scoring framework. Both the instructors and DeepSeek applied identical scoring criteria and domain-specific descriptors to ensure consistency in assessment. At the conclusion of the semester, participants were asked to write an argumentative essay on a given topic within a 30-min period. The questionnaire, which encompassed all relevant scales and consent forms, was uploaded to a Chinese online survey platform,^1^ and the corresponding quick response (QR) code was generated for access. Subsequent to notifying the students of their final scores, we requested the instructors to disseminate the study’s introduction and gave the QR code to the students in the class WeChat group. Students were advised of their right to participate voluntarily and the confidentiality of their responses. Students were able to scan the QR code using their mobile phones to access the online questionnaire, which took approximately 15–20 min to complete.

### 3.4 Data analysis

Initial data screening, including descriptive statistics and normality tests, was conducted in SPSS 28.0. After standard multiple regression analyses addressing the first two research questions, the proposed parallel mediation model was tested using PROCESS v2.16.3 (Model 4; Hayes, 2022). Consistent with contemporary mediation practice, the analysis focused on the estimation of indirect effects rather than traditional causal-steps procedures (Zhao et al., 2010). Model 4 enables the simultaneous estimation of multiple mediators, allowing for the assessment of direct and specific indirect effects while controlling for shared variance. Bias-corrected bootstrapping was applied to generate confidence intervals, as resampling methods provide more accurate and robust estimates of indirect effects than normal theory-based approaches (MacKinnon et al., 2004; Preacher and Hayes, 2008). Given that the study aimed to compare the independent mediating roles of FLWE and FLWB, this modelling approach was methodologically appropriate.

## 4 Results

Table 2 presents the descriptive statistics and inter-correlations among ILWS, OLWS, FLWE, FLWB, and WA. Overall, participants reported relatively high levels of ILWS (*M* = 25.70, *SD* = 4.81) and OLWS (*M* = 24.02, *SD* = 4.90), suggesting that they both aspired to improve their English writing and perceived certain external expectations regarding their performance. With respect to emotional experiences, students reported higher levels of FLWE (*M* = 33.21, *SD* = 6.14) than FLWB (*M* = 13.52, *SD* = 5.44), indicating that positive emotions were more salient than negative ones during the writing process. Writing achievement scores were also relatively high (*M* = 82.65, *SD* = 5.99). The results for all parameters were within the acceptable ranges of −2 to +2 for Skewness and −7 to +7 for Kurtosis (West et al., 1995), showing normal distribution of the variables, hence permitting following parametric studies. Correlational analyses showed that both ideal L2 writing self (ILWS) and ought-to L2 writing self (OLWS) were positively associated with foreign language writing enjoyment (FLWE) and writing achievement (WA), and negatively associated with foreign language writing boredom (FLWB). Moreover, FLWE was positively correlated with WA, whereas FLWB was negatively correlated with WA, providing preliminary support for the hypothesized mediation relationships.

**Table 2:** Descriptive statistics and bivariate correlations between variables.

Variable	ILWS	OLWS	FLWE	FLWB	WA
ILWS	–				
OLWS	0.609**	–			
FLWE	0.547**	0.438**	–		
FLWB	−0.503**	−0.328**	−0.680**	–	
WA	0.535**	0.374**	0.534**	−0.488**	–
Mean (*SD*)	25.70 (4.81)	24.02 (4.90)	33.21 (6.14)	13.52 (5.44)	82.65 (5.99)
Skewness	−1.29	−0.88	−0.20	0.11	−0.84
Kurtosis	2.54	1.62	0.04	−0.81	0.42

ILWS, ideal L2 writing self; OLWS, ought-to L2 writing self; FLWE, foreign language writing enjoyment; FLWB, foreign language writing boredom; SD, standard deviation; ^**^p < 0.01.

To further examine the predictive relationships, parallel mediation analyses were conducted using PROCESS Model 4 with 10,000 bootstrap samples. As shown in Table 3, when ILWS was entered as the predictor, it significantly predicted writing achievement in the total effect model (*B* = 0.668, *SE* = 0.057, *p* < 0.001; *β* = 0.536), indicating that stronger ideal L2 writing self was associated with higher achievement. ILWS also significantly predicted FLWE positively (*β* = 0.547) and FLWB negatively (*β* = −0.503). After FLWE and FLWB were simultaneously included in the model, the direct effect of ILWS on WA decreased but remained significant (*B* = 0.396, *SE* = 0.065, *p* < 0.001; *β* = 0.318), suggesting partial mediation. Within this full model, FLWE positively predicted WA (*β* = 0.255), whereas FLWB negatively predicted WA (*β* = −0.155). The overall model explained 38.2% of the variance in writing achievement.

**Table 3:** Bootstrap estimates of parallel mediation effects of ILWS on writing achievement (*N* = 349).

Effect type	Pathway	*B*	*β*	BootSE	95% BC CI	Proportion of total effect
Total effect (*c*)	ILWS → WA	0.668	0.536	0.057	[0.557, 0.779]	—
Direct effect (*c*′)	ILWS → WA (controlling for FLWE & FLWB)	0.396	0.318	0.065	[0.270, 0.523]	—
Total indirect effect	ILWS → WA (via FLWE & FLWB)	0.271	0.218	0.050	[0.179, 0.374]	40.6%
Specific indirect effect	ILWS → FLWE → WA	0.174	0.140	0.061	[0.057, 0.294]	26.0%
Specific indirect effect	ILWS → FLWB → WA	0.097	0.078	0.053	[0.001, 0.209]	14.6%
Contrast (C1)	FLWE − FLWB	0.077	0.061	0.103	[−0.130, 0.274]	—

Indirect effects are reported as unstandardized coefficients (*B*) with bias-corrected bootstrap 95% confidence intervals (10,000 resamples). Indirect effects are considered significant when the confidence interval does not include zero. Proportion mediated was calculated as the indirect effect divided by the total effect. Standardized coefficients (*β*) represent effect sizes and allow for comparison across paths.

**Figure 1:**
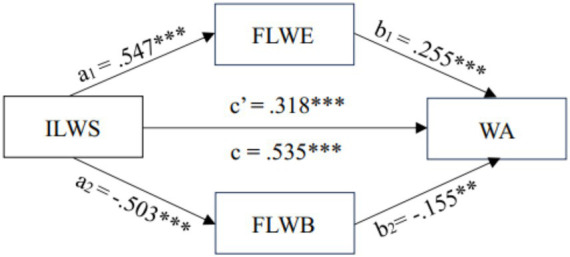
The statistical diagram of parallel multiple mediation Model 1 **p < 0.01, ***p < 0.001.

Bootstrap analyses revealed that the total indirect effect of ILWS on WA through FLWE and FLWB was significant [*B* = 0.271, 95% *CI* (0.179, 0.374)], accounting for approximately 40.6% of the total effect, with both specific indirect effects reaching statistical significance (see Table 3). Specifically, the indirect effect through FLWE was significant [*B* = 0.174, 95% *CI* (0.057, 0.294)], indicating that students with stronger ideal writing selves tended to experience greater enjoyment, which in turn enhanced their writing achievement. The indirect effect through FLWB was also significant [*B* = 0.097, 95% *CI* (0.001, 0.209)], suggesting that reduced boredom partially transmitted the influence of ILWS on achievement. However, the difference between the two indirect effects was not statistically significant, as the confidence interval for their contrast included zero, implying that neither pathway was significantly stronger than the other. The standardized path coefficients for the parallel mediation model with ILWS as the predictor are illustrated in Figure 1.

A similar pattern emerged when ought-to L2 writing self (OLWS) was examined as the predictor. As displayed in Table 4, OLWS significantly predicted writing achievement in the total effect model (*B* = 0.458, *SE* = 0.061, *p* < 0.001; *β* = 0.374). It also positively predicted FLWE (*β* = 0.438) and negatively predicted FLWB (*β* = −0.328). When both mediators were entered simultaneously, the direct effect of OLWS on WA was reduced but remained statistically significant (*B* = 0.202, *SE* = 0.060, *p* < 0.01; *β* = 0.165), again indicating partial mediation. In the full model, FLWE positively predicted WA (*β* = 0.310), whereas FLWB negatively predicted WA (*β* = −0.224). The model accounted for 33.7% of the variance in writing achievement.

**Table 4:** Bootstrap estimates of parallel mediation effects of OLWS on writing achievement (*N* = 349).

Effect type	Pathway	*B*	*β*	BootSE	95% BC CI	Proportion of total effect
Total effect (*c*)	OLWS → WA	0.458	0.374	0.061	[0.338, 0.578]	—
Direct effect (*c*′)	OLWS → WA (controlling for FLWE & FLWB)	0.202	0.165	0.060	[0.085, 0.320]	—
Total indirect effect	OLWS → WA (via FLWE & FLWB)	0.256	0.209	0.054	[0.157, 0.370]	55.9%
Specific indirect effect	OLWS → FLWE → WA	0.166	0.136	0.055	[0.070, 0.283]	40.3%
Specific indirect effect	OLWS → FLWB → WA	0.090	0.073	0.038	[0.026, 0.174]	19.6%
Contrast (C1)	FLWE − FLWB	0.077	0.063	0.077	[−0.076, 0.233]	—

Indirect effects are reported as unstandardized coefficients (*B*) with bias-corrected bootstrap 95% confidence intervals (10,000 resamples). Indirect effects are considered significant when the confidence interval does not include zero. Proportion mediated was calculated as the indirect effect divided by the total effect. Standardized coefficients (*β*) represent effect sizes and allow for comparison across paths.

**Figure 2:**
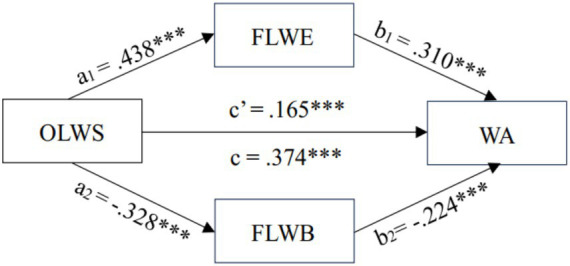
The statistical diagram of parallel multiple mediation Model 2 ***p < 0.001.

The bootstrap results further demonstrated that the total indirect effect of ought-to L2 writing self on writing achievement via foreign language writing enjoyment and boredom was significant [*B* = 0.256, 95% *CI* (0.157, 0.370)], representing 55.9% of the total effect, with both specific indirect effects reaching statistical significance (see Table 4). The indirect effect through foreign language writing enjoyment was significant [*B* = 0.166, 95% *CI* (0.070, 0.283)], as was the indirect effect through foreign language writing boredom [*B* = 0.090, 95% *CI* (0.026, 0.174)]. Although the indirect effect via writing enjoyment appeared numerically larger than that via writing boredom, the contrast between the two pathways was not statistically significant. Taken together, these findings indicate that both positive and negative writing-related emotions function as parallel mediators linking future L2 writing selves to writing achievement, with enjoyment showing a consistently stronger, though not significantly different, contribution. The standardized path coefficients for the parallel mediation model with ought-to L2 writing self as the predictor are presented in Figure 2.

## 5 Discussion

The findings of this study illuminate the intricate relationships between foreign language writing selves (FL2WS), EFL writing emotions (FLWE and FLWB), and writing achievement. Consistent with the L2MSS (Dörnyei and Chan, 2013) and Higgins’ (1987) self-discrepancy theory, the results indicate that both ideal L2 writing self (ILWS) and ought-to L2 writing self (OLWS) positively predict foreign language writing enjoyment (FLWE) while negatively predicting foreign language writing boredom (FLWB). Specifically, the positive influence of ILWS on enjoyment aligns with previous research (Tahmouresi and Papi, 2021; Yao et al., 2024), suggesting that the narrowing of the gap between the ideal and actual L2 writing self enhances students’ emotional satisfaction. Furthermore, ILWS was found to negatively predict FLWB, reinforcing the argument that learners driven by aspirations for success exhibit a promotion focus (Struk et al., 2016). Even when facing demanding writing tasks, these positive aspirations may encourage learners to adopt active responses to moments of disengagement, suggesting that a vivid ideal self-image is associated with a more favorable emotional profile in writing. These findings should be understood as complementing, rather than displacing, the established literature on writing apprehension and anxiety by bringing enjoyment and boredom more explicitly into writing-specific motivational-emotional models.

A significant contribution of this research is the finding that ought-to L2 writing self (OLWS) positively predicted foreign language writing enjoyment (FLWE) and negatively predicted foreign language writing boredom (FLWB), a pattern that differs from earlier studies by Amini et al. (2023) and Tahmouresi and Papi (2021). One possible explanation lies in the distinction between the self-perceived ought-to L2 writing self (ought-to L2 self/own) and the externally influenced version (ought-to L2 self/other). As Papi et al. (2019) proposed in their 2 × 2 model, ought-to L2 self/own may reflect a more self-relevant sense of responsibility than forms of ought-to motivation shaped primarily by external expectations. In the present context, where English writing is closely tied to academic requirements, some writing-related obligations may have been experienced by learners as personally meaningful rather than as purely external pressure. This may help explain why OLWS/own was associated with greater enjoyment and lower boredom in the present study. At the same time, this interpretation should be treated with caution, since the study did not directly examine the psychological process through which such obligations may have been internalized. Other explanations, such as stronger academic discipline, greater familiarity with exam-oriented writing tasks, or sensitivity to institutional expectations, may also account for the observed pattern. In this sense, boredom may reflect not only disengagement but also learners’ responses to how writing demands are appraised and managed (Csizér and Lukács, 2010).

These findings may be better understood not only in relation to cross-context differences, but also in light of broader work on L2 motivation, regulatory focus, and writing-specific emotions. From this perspective, the present results add to a growing literature suggesting that the motivational significance of the ought-to self may depend on how it is framed, experienced, and embedded in particular learning environments. The comparison with Tahmouresi and Papi’s (2021) Iranian sample remains informative, but it should not be overextended as the sole explanation. Other factors may also have contributed, including participants’ developmental stage, the exam-oriented nature of the writing context, and the use of writing-specific emotional measures in the present study. Taken together, these considerations suggest that the role of OLWS may be more context-sensitive and theoretically nuanced than accounts that treat the ought-to self as uniformly maladaptive.

An additional point that merits discussion concerns the relationship between participants’ self-perceived English proficiency and their objectively assessed performance. In the present sample, the mean placement-test score was 71 out of 100, whereas self-perceived English proficiency was 5.51 on a 10-point scale, suggesting not a sharp mismatch but a relatively cautious self-appraisal. This pattern is consistent with prior cross-cultural research showing that Asian learners may report lower self-efficacy or more modest competence beliefs despite performing comparatively well academically (Eaton and Dembo, 1997; Yeung et al., 2024). Eaton and Dembo (1997), for instance, found that Asian American students reported lower self-efficacy yet outperformed their non-Asian peers, and that fear of academic failure better explained their achievement behavior than self-efficacy. Similarly, Yeung et al. (2024) noted that among Chinese learners, self-efficacy may play a less prominent role than apprehension or failure-related concerns in predicting writing performance, partly because Chinese students are often socialized to value effort, obligation, and persistence. From this perspective, the relatively conservative self-evaluations observed in the present study do not contradict the prominence of the ought-to L2 writing self. Rather, they may help explain why responsibility-based self-guides remain salient even when students’ objective performance is acceptable: learners may still perceive themselves as not yet fully adequate relative to academic expectations and possible negative consequences. At the same time, this interpretation should be made cautiously, since self-perceived proficiency was included only as a background variable and was not modeled as a predictor, mediator, or moderator in the present study. Future research could therefore examine more directly whether discrepancies between perceived and actual proficiency shape the strength of future L2 writing selves and their emotional and achievement-related consequences.

Beyond emotional outcomes, this study confirms that both ideal L2 writing self (ILWS) and ought-to L2 writing self (OLWS) are robust predictors of writing achievement. While the influence of ILWS—an aspirational, promotion-oriented vision—is well-documented (Jang and Lee, 2019; Tang et al., 2024), the significant positive role of OLWS observed here is particularly noteworthy. Previous literature has often critiqued the limited predictive validity of the ought-to self (Al-Hoorie, 2018). However, as Markus and Nurius (1986) argued, “possible selves” are most effective when they are personally salient. In the present study, pressure to avoid negative academic outcomes (e.g., “I must learn how to write; otherwise, I won’t get my degree”) may have functioned as a meaningful source of motivation. This prevention focus ensures that students achieve the “minimal goals” necessary for success (Higgins, 1998). Thus, the OLWS/own serves as a key driver for avoiding undesirable outcomes, guiding students toward better performance alongside the aspirational pull of the ILWS.

Regarding the underlying psychological mechanism, the results highlight the predictive roles of FLWE and FLWB in relation to achievement, consistent with Pekrun’s (2006) CVT. Achievement emotions are closely associated with motivation, sustained attention, and cognitive resource availability. Positive emotions such as enjoyment are generally associated with broader engagement and more adaptive involvement in writing, whereas boredom has more often been linked to reduced focus and weaker engagement in writing-specific research (Botes et al., 2022; Li and Li, 2024). At the same time, as noted earlier, boredom need not be viewed as uniformly maladaptive in every instance; under some conditions, it may also function as an affective signal that learners’ current engagement with the task is suboptimal, thereby prompting effort regulation or strategic adjustment (Danckert and Elpidorou, 2023). From a control-value perspective, boredom may arise when writing tasks are perceived as either far beyond or below learners’ capabilities, suggesting that at least some of its antecedents may be responsive to changes in task design, challenge level, or instructional support (Tze et al., 2016). Our co-mediation model suggests that future L2 writing selves relate to achievement partly through these emotional experiences. More broadly, the present findings contribute to the wider literature by suggesting that writing-related motivation and writing emotions are better understood as jointly operating processes rather than as separate predictors of achievement, and that the role of the ought-to self may be more differentiated in writing-specific contexts than has often been assumed in general L2 motivation research. Learners with higher levels of ideal or ought-to L2 writing selves may be better positioned to respond to such emotional cues in ways that sustain or restore engagement during writing. In this respect, the present findings are consistent with the possibility that responsibility-based motivation may, in some writing-specific contexts, function in a more adaptive manner than has often been assumed. At the same time, the underlying mechanism should not be overstated, as the present study did not directly examine how such responsibility was psychologically experienced or regulated.

At the same time, the use of the end-of-semester writing examination as the outcome measure calls for cautious interpretation. All participants completed a 12-week writing course prior to the examination. Instruction was delivered under a unified syllabus with synchronized pacing across sections, and students completed multiple guided and timed practice essays. Consequently, the writing scores likely reflected not only learners’ motivational and emotional dispositions but also the effects of prior instruction, classroom alignment with the exam, and familiarity with the assessment format. This does not necessarily reduce the value of the outcome measure. Rather, it indicates that the writing scores should be understood within the instructional context in which they were produced. In this sense, the present findings are best interpreted as explaining writing achievement in an instructed, exam-oriented EFL setting, rather than writing ability in a fully decontextualized sense. Moreover, since all participants shared the same curricular environment and received broadly comparable instructional preparation, the relationships observed among motivation, emotion, and achievement remain interpretable within this common pedagogical framework.

In a nutshell, this study underscores the necessity of a nuanced understanding of L2 self-guides within domain-specific contexts. By integrating the L2MSS with CVT, we demonstrate that both the “ideal” vision of a fluent writer and the “ought-to” sense of academic responsibility are essential catalysts for success. In high-stakes, examination-oriented settings, OLWS/own may function not only as a source of pressure, but also as a potentially adaptive emotional and motivational resource. The present findings are consistent with the possibility that some learners may respond to writing-related obligations in ways that help them navigate the emotional challenges of L2 writing more adaptively, including responding more constructively to experiences of boredom, thereby supporting stronger academic outcomes.

## 6 Conclusion

The present study adds to the growing body of research on the dynamic interaction between motivation and emotion in Asia-Pacific foreign language learning. By integrating the L2 Motivational Self System and Control-Value Theory, the findings show that future-oriented writing self-guides are associated with writing-related emotional experiences, which in turn are linked to writing achievement. In this way, the study offers a more fine-grained account of how motivational and emotional factors may jointly shape L2 writing performance in an exam-oriented EFL context.

A key contribution of this research lies in its treatment of the ought-to L2 writing self. While this self-guide has often been viewed in the literature as primarily pressure-laden or less adaptive, the present findings suggest that, in this context, OLWS/own may also function as a potentially constructive motivational resource. More specifically, its positive association with writing enjoyment and negative association with writing boredom is consistent with the possibility that some forms of writing-related obligation were experienced as self-relevant responsibilities rather than purely external pressure. At the same time, this interpretation should be made cautiously, because the study examined associations among variables rather than the psychological process through which such responsibilities may have been internalized.

Overall, the findings underscore the importance of examining both ideal and ought-to dimensions of future L2 writing selves within domain-specific and socioculturally situated contexts. They also suggest that motivation and emotion in L2 writing are better understood as interconnected processes rather than isolated predictors of achievement. By extending attention beyond anxiety to include enjoyment and boredom, the study contributes to a more differentiated understanding of the psycho-affective processes involved in foreign language writing.

## 7 Implication

This research has practical implications for English as a Foreign Language (EFL) writing courses in private higher education institutions. At the program level, school administrators may take students’ writing proficiency into account when making placement decisions, as a more balanced grouping structure may help teachers respond more effectively to learners’ instructional and emotional needs. Although the present study did not directly examine placement effects, the findings suggest that classroom contexts that are more responsive to students’ current writing level may be more conducive to positive emotional experiences during writing.

At the classroom level, the findings underscore the importance of systematically cultivating students’ future L2 writing selves while attending to their emotional experiences during writing. Because both the ideal and ought-to L2 writing selves were positively associated with writing achievement in the present study, writing instructors may benefit from helping students form clearer and more personally meaningful future writing self-images. In practice, this may involve brief vision-building activities, such as asking students to articulate what kind of English writer they hope to become, set short-term writing goals, or reflect on how writing development relates to their academic progress and future professional needs (Dörnyei and Ryan, 2015; Tahmouresi and Papi, 2021).

The findings also suggest that external writing-related expectations may be more effective when students are encouraged to experience them as more personally meaningful and self-relevant, rather than merely as outside pressure (Papi et al., 2019). In this respect, teachers may help students see writing not only as a course requirement, but also as an important component of their broader academic development. For example, instructors may make explicit how writing competence supports coursework, degree completion, and future study or employment, thereby encouraging students to reinterpret external expectations as personally meaningful responsibilities.

In addition, since writing enjoyment and boredom were both associated with writing achievement, EFL writing instruction should pay closer attention to students’ emotional experiences in the writing process. One practical way to do so is to assign writing tasks at an appropriate level of difficulty, so that students are neither overwhelmed nor insufficiently challenged. Teachers may also help reduce boredom and sustain engagement by varying task topics, allowing limited choice in content or examples, breaking extended writing tasks into manageable stages, and providing timely feedback during the writing process rather than only after task completion. Such practices may help make writing more enjoyable, reduce boredom, and support students’ writing development (Li et al., 2023).

Taken together, these findings suggest that EFL writing instruction can better support students’ motivational development and emotional balance by working simultaneously on their future self-concepts and their classroom writing experiences.

## 8 Limitations and future research

When evaluating the findings, it is important to take into account a number of limitations. First, concentrating just on students from a single Chinese private university restricts the applicability of the findings to other educational settings. While the sample size of 349 is substantial within this context, it is not necessarily representative of all Chinese EFL learners. The specific demographic and institutional characteristics of this private university, such as its focus on tuition-based revenue and Sino-foreign collaborations, may influence students’ motivational and emotional experiences in ways that differ from those in public institutions or other regions. Future research should consider taking into consideration samples from a wider range of institutions, including private and public universities or schools from different regions, to assess the broader applicability of these findings to the Chinese EFL context and beyond.

Second, the assessment of future L2 writing selves only addressed the “own” perspective, neglecting the “other” dimension, which is theoretically significant in the 2 × 2 paradigm (Papi et al., 2019). Third, the cross-sectional design prevents the analysis of the evolution of these motivational and emotional variables during students’ writing development. In addition, writing achievement was assessed through an end-of-semester examination conducted after a semester of instruction. As such, the outcome scores may have been shaped not only by students’ motivational and emotional dispositions but also by instructional alignment, repeated practice with similar task types, and familiarity with the assessment format. Future research may therefore benefit from including a wider range of writing outcomes, such as less instructionally aligned tasks, transfer tasks, or longitudinal performance measures, to test whether the observed relationships generalize beyond the immediate classroom assessment context.

These constraints indicate multiple intriguing avenues for forthcoming research. In response to Li et al.'s (2023) request for evidence from various student cohorts, research should investigate these correlations within distinct institutional and cultural frameworks. Subsequent research should focus on creating complete scales that encompass all aspects of the 2 × 2 self-guides model, preferably utilizing qualitative methodologies that consider contextual variables (Papi et al., 2019). Furthermore, expanding upon Li's (2021) focus on contextual antecedents, research might examine how particular instructional elements (e.g., feedback methodologies, task design) elicit the control-value appraisals that generate writing emotions. Longitudinal designs would be especially beneficial for comprehending the dynamic interaction between future selves and emotions during various learning stages. In addition, although the present study deliberately focused on enjoyment and boredom, future research may usefully examine how these emotions operate alongside more established constructs such as writing apprehension or writing anxiety within the same model. Broadening this line of inquiry to other language skills may also contribute to a more comprehensive understanding of motivational-emotional processes in L2 learning.

## Data Availability

The raw data supporting the conclusions of this article will be made available by the authors, without undue reservation.

## Footnotes

1
https://www.wjx.cn/
